# Breast Cancer Stem Cell-Like Cells Are More Sensitive to Ionizing Radiation than Non-Stem Cells: Role of ATM

**DOI:** 10.1371/journal.pone.0050423

**Published:** 2012-11-21

**Authors:** Seog-Young Kim, Juong G. Rhee, Xinxin Song, Edward V. Prochownik, Douglas R. Spitz, Yong J. Lee

**Affiliations:** 1 Department of Surgery, School of Medicine, University of Pittsburgh, Pittsburgh, Pennsylvania, United States of America; 2 The Radiation Oncology Research Laboratory, Department of Radiation Oncology, University of Maryland School of Medicine, Baltimore, Maryland, United States of America; 3 Department of Pediatrics, School of Medicine, University of Pittsburgh, Pittsburgh, Pennsylvania, United States of America; 4 Department of Molecular Genetics and Biochemistry, School of Medicine, University of Pittsburgh, Pittsburgh, Pennsylvania, United States of America; 5 Free Radical and Radiation Biology Program, Department of Radiation Oncology, Holden Comprehensive Cancer Center, The University of Iowa, Iowa City, Iowa, United States of America; Dana-Farber/Harvard Cancer Institute, United States of America

## Abstract

There are contradictory observations about the different radiosensitivities of cancer stem cells and cancer non-stem cells. To resolve these contradictory observations, we studied radiosensitivities by employing breast cancer stem cell (CSC)-like MDA-MB231 and MDA-MB453 cells as well as their corresponding non-stem cells. CSC-like cells proliferate without differentiating and have characteristics of tumor-initiating cells [1]. These cells were exposed to γ-rays (1.25–8.75 Gy) and survival curves were determined by colony formation. A final slope, D_0_, of the survival curve for each cell line was determined to measure radiosensitivity. The D_0_ of CSC-like and non-stem MDA-MB-453 cells were 1.16 Gy and 1.55 Gy, respectively. Similar results were observed in MDA-MB-231 cells (0.94 Gy vs. 1.56 Gy). After determination of radiosensitivity, we investigated intrinsic cellular determinants which influence radiosensitivity including cell cycle distribution, free-radical scavengers and DNA repair. We observed that even though cell cycle status and antioxidant content may contribute to differential radiosensitivity, differential DNA repair capacity may be a greater determinant of radiosensitivity. Unlike non-stem cells, CSC-like cells have little/no sublethal damage repair, a low intracellular level of ataxia telangiectasia mutated (ATM) and delay of γ-H2AX foci removal (DNA strand break repair). These results suggest that low DNA repair capacity is responsible for the high radiosensitivity of these CSC-like cells.

## Introduction

Breast cancer is the most common cancer in American women, and the second leading cause of cancer death [2], [3]. Due to improvement of early diagnosis with mammography and the development of more effective adjuvant therapies including radiation, the past 20 years have seen a significant decrease in mortality from breast cancer in the United States and elsewhere [3]. However, many women still suffer recurrence and incurable metastases, and the optimal management of these diseases remains undefined.

Ionizing radiation and chemotherapeutic agents continue to be a frontline therapy for local control of breast cancer where surgery is either not possible or undesirable such as in breast conservation therapy. Previous studies suggest that the failure of conventional therapy is due to cancer stem (tumor-initiating) cells which are inherently resistant to radiation and chemotherapeutic agents [4]–[7]. Bao et al. [4] reported that their radioresistance is mediated through preferential activation of the DNA damage checkpoint response and an increase in DNA repair capacity. However, Ropolo et al. [8] claimed that cell cycle distribution and intracellular level of activated checkpoint proteins rather than DNA repair capacity contribute to the intrinsic radioresistant property of cancer stem cells (CSCs). Nevertheless, recent studies reveal that CSC may be more sensitive to radiation, rather than radioresistant, compared with established cancer cell lines [9]–[11]. These discrepancies are probably due to dynamic properties of CSCs as well as limitations of experimental analytical techniques.

**Figure 1:**
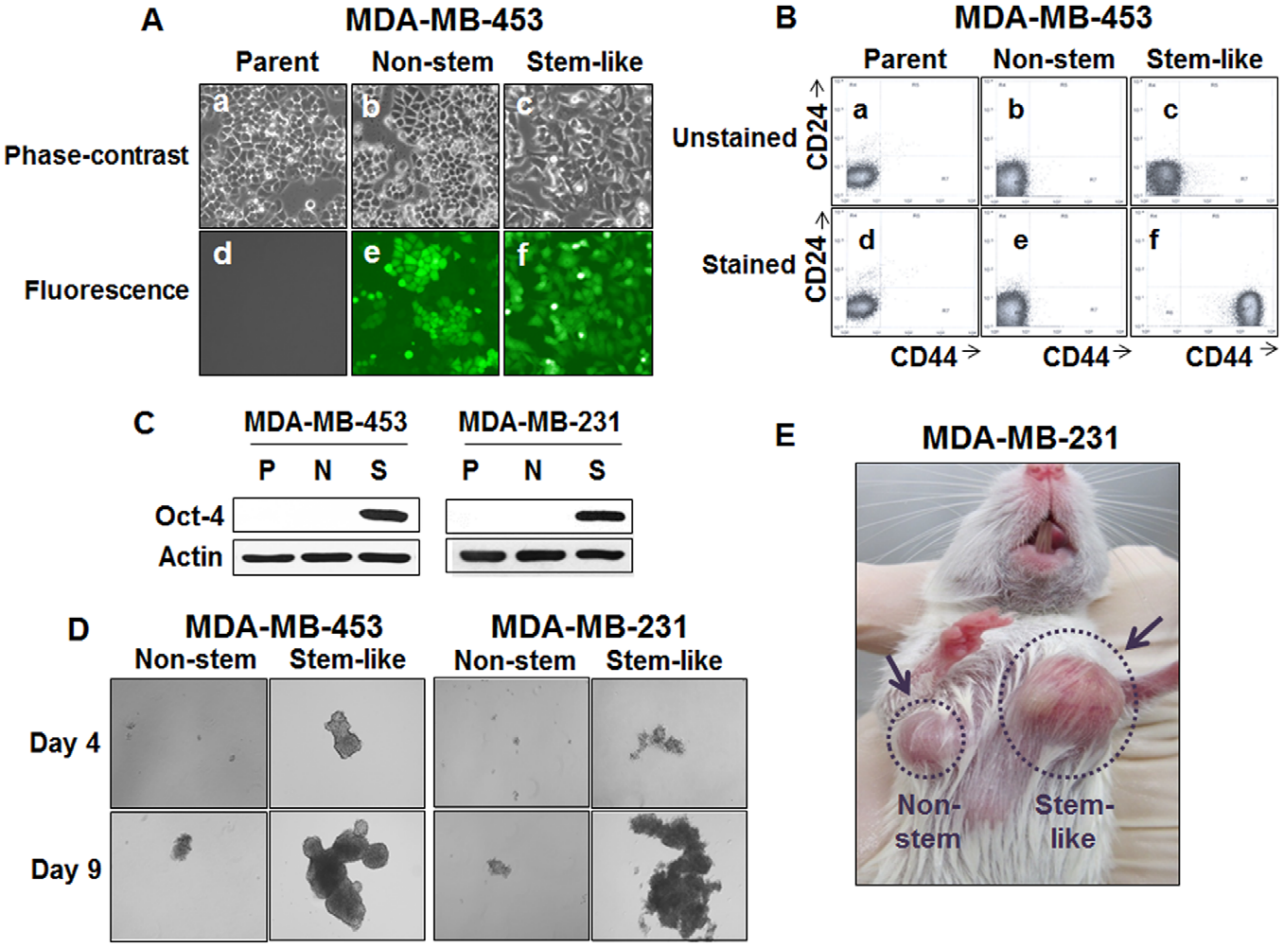
Characterization of breast cancer stem cell-like (CSC-like) cells. (**A**) Parental MDA-MB-453 cells were transfected with a plasmid encoding GFP under the control of the CMV promoter (non-stem) or Oct3/4 promoter (stem-like). After selection in G-418, GFP+ colonies were pooled. Phase-contrast images and fluorescence images of parental cells (Parent: a, d), CMV-GFP-transfected non-stem cells (Non-stem: b, e) or Oct3/4-GFP-transfected CSC-like cells (Stem-like: c, f) were visualized by light (**Phase-contrast: a–c**) or UV (**Fluorescence: d–f**) microscopy. (**B**) Flow cytometry characterization of parental, non-stem, or CSC-like cells was performed. CMV promoter-driven GFP cDNA (**e;** non-stem cells) or human Oct3/4 promoter-driven GFP cDNA (**f;** stem-like cells) transfected MDA-MB-453 cells were stained with surface marker antibodies (CD24, CD44) and evaluated by flow cytometry. (**a–c**) Unstained cells and (**a, d**) parental cells. (**C**) Stem cell-associated Oct-4 gene expression was examined in MDA-MB-453 and MDA-MB-231 parental (P), non-stem (N) and CSC-cell like (S) cells. Cells were harvested with lysis buffer. Lysates containing equal amounts of protein (20 µg/ml) were separated by SDS-PAGE, and immunoblotted with anti-Oct-4 antibody. Actin was shown as an internal standard. (**D**) Mammosphere formation was compared in MDA-MB-231 and MDA-MB-453 CSC-like and non-stem cells. For mammosphere formation, 1,000 cells from stem-like cells or non-stem cells were plated into ultra-low attachment plates. Phase-contrast images of mammospheres of non-stem (left panels) or CSC-like (right panels) cells were obtained 4 days or 9 days later. (**E**) Xenograft tumor formation was established with CSC-like and non-stem MDA-MB-231 cells. For tumor formation in NOD/SCID mice, 1×10^4^ stem-like or non-stem cells were injected into the upper mammary fat pad. Tumor volumes were measured 30 days after injection.

Breast CSCs have been well studied. The results of both Al-Hajj and colleagues and Ponti and colleagues suggest that breast cancer cells with the capacity for long-term self-renewal are enriched within the CD44^+^ (hyaluronan receptor), CD24^−^ (P-selectin), and ESA^+^ (epithelial surface antigen) subset [12], [13]. Because these breast CSCs are only a small portion (0.1–5%) of the population, it is extremely difficult to perform biochemical analysis and colony formation assay with CSCs. To resolve this difficulty, we employed permanently blocked cancer stem cells derived from two breast cancer cell lines. As previously described, CSC-like cells and their corresponding non-stem cells were generated by stable transfection of green fluorescent protein (GFP) under the control of the human octamer binding transcription factor 3/4 promoter (Oct3/4) and cytomegalovirus (CMV) promoter, respectively [1]. Interestingly, these CSC-like cells can proliferate without differentiation, have characteristics of tumor-initiating cells and express tumor cell markers (CD44^+^ and CD24^−^) characteristic of CSCs [1]. These CSC-like cells and their isogenic non-CSC lines allow us to perform quantitative clonogenic survival assay and biochemical analysis.

In this study we observed that CSC-like cells were more sensitive to ionizing radiation than their corresponding subset non-stem cells. Our data suggest that the lower levels of ATM in the CSC-like cells likely explain their intrinsic radiosensitivity.

**Figure 2:**
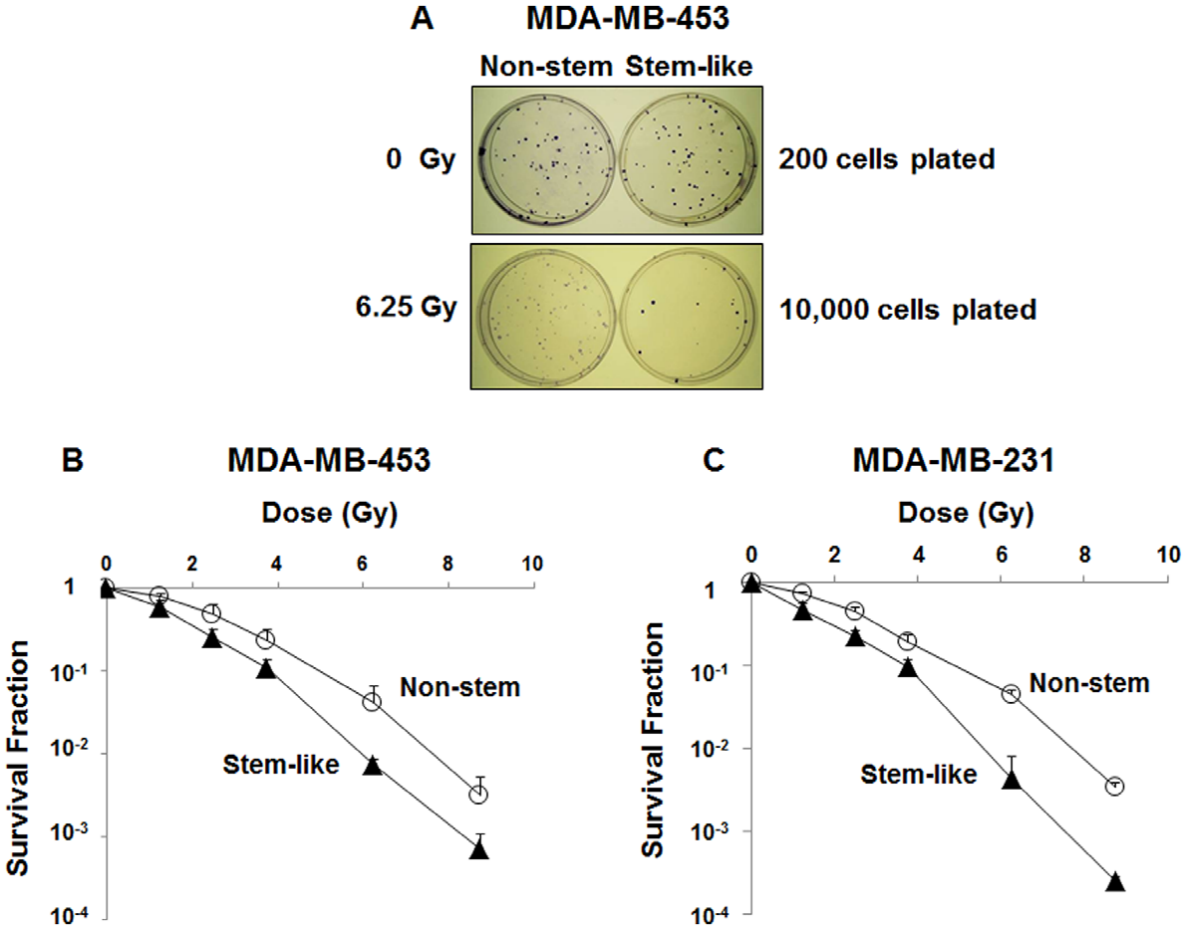
Survival curves for non-stem and CSC-like MDA-MB-453 and MDA-MB-231 cells after irradiation. (**A**) Colonies were obtained with non-stem and stem-like MDA-MB-453 cells. Cells were unirradiated (0 Gy) or irradiated (6.25 Gy), trypsinized, counted and plated. Cells were grown for 1–3 weeks and stained with crystal violet. (**B, C**) Survival curves for non-stem and stem-like MDA-MB-453 (**B**) and MDA-MB-231 (**C**) cells were determined after irradiation. Cells were exposed to various doses (1.25 Gy-8.75 Gy) of γ-radiation. Cells were trypsinized, counted and plated. Colony formation was determined 1–3 weeks after irradiation. Error bars represent standard error from the mean for three separate experiments.

## Materials and Methods

### Cell Culture

Permanently blocked cancer stem cell (CSC)-like MDA-MB-453 and MDA-MB-231 cell lines were generated as previously described following stable transfection with a human Oct3/4 promoter driving the expression of green fluorescent protein (GFP) [1]. In brief, when cells were transfected with plasmids containing Oct3/4 promoter-driven GFP, G-418-resistant colonies were pooled and GFP-positive and GFP-negative cells were separated using a flow cytometer. GFP-positive cells were maintained in G418-containing DMEM or **RPMI**. GFP-positive cells were periodically subjected to flow cytometry to evaluate the fraction of GFP-positive cells. When cells were stably transfected with these plasmids, unexpectedly, these GFP-positive CSC-like cells were unable to differentiate and remained blocked in a CSC-like state. The mechanism still remains unknown of how permanently blocked CSC-like cells can be derived from breast cancer cell lines by expressing Oct3/4 promoter-driven GFP. As a control, the corresponding non-CSC populations were generated by expressing GFP under the control of a CMV immediate-early promoter. The CSC-like cells can proliferate without differentiation and have characteristics of tumor-initiating cells. These cells were cultured in DMEM or RPMI 1640 with 10% FBS (HyClone, Logan, UT, USA) and 26 mM sodium bicarbonate for the monolayer cell culture. Petri-dishes containing cells were kept in a 37°C humidified incubator with a mixture of 95% air and 5% CO_2_.

### Mammosphere Formation

MDA-MB-231 or MDA-MB-453 CSC-like cells or non-stem cells were placed in ultra-low attachment 24 well culture plates (Corning, Lowell, MA, USA) for mammosphere formation assay.

**Figure 3:**
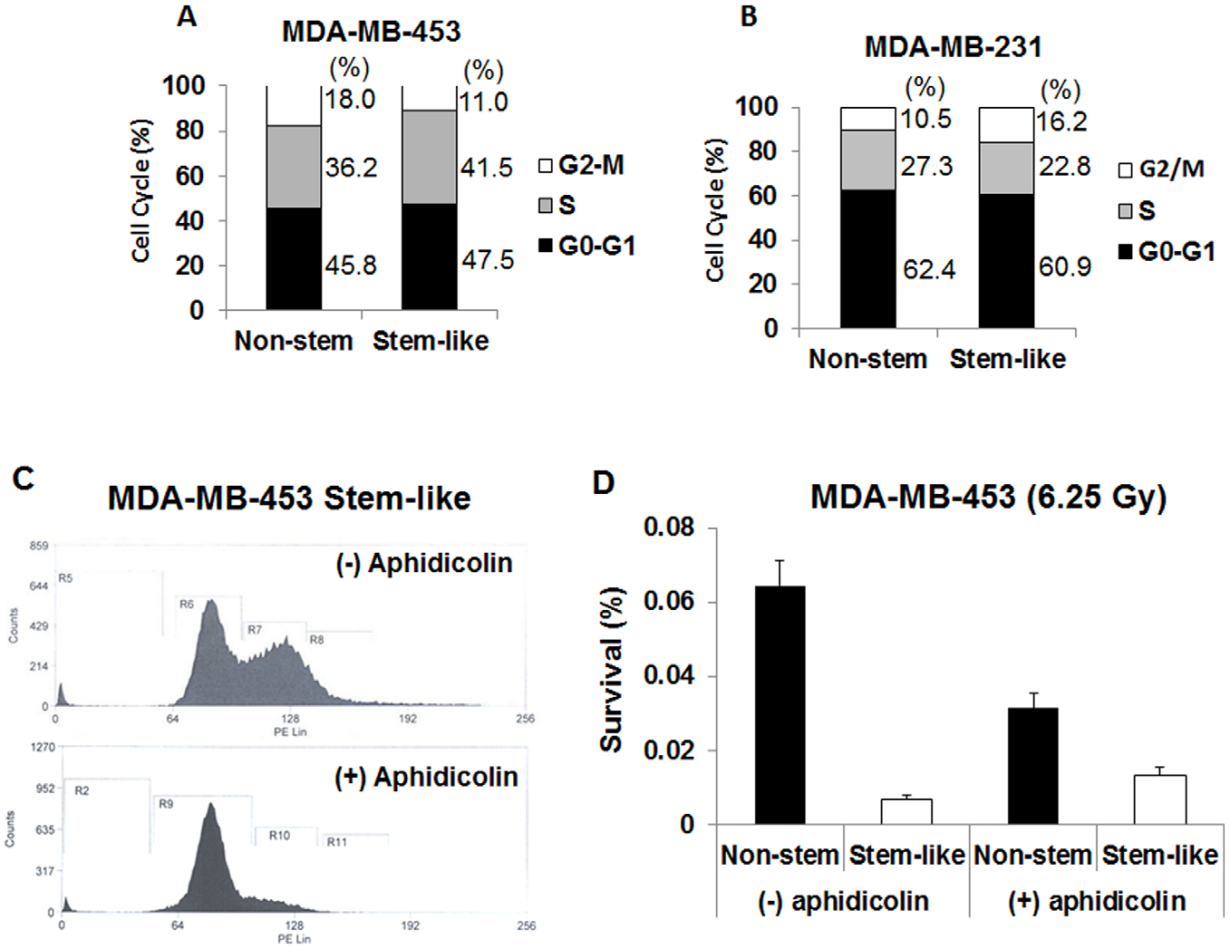
Flow cytometry analysis of cell cycle in asynchronous and effect of aphidicolin treatment on phase distribution and radiosensitivity in non-stem and CSC-like cells. Flow cytometry was performed on non-stem and stem-like MDA-MB-453 cells (**A**) or MDA-MB-231 cells (**B**). The percentages of cells in the G_2_/M, S, and G_1_ were analyzed and plotted. Non-stem and CSC-like MDA-MB-453 cells were treated with aphidicolin (5 µM) for 16 hr. After aphidicolin treatment, cell cycle was analyzed (**C**) and radiation sensitivity was determined by colony formation after irradiation (6.25 Gy) (**D**). Error bars represent standard error from the mean for three separate experiments.

### Xenograft Tumor Formation

MDA-MB-231 CSC-like cells or non-stem cells (1×10^4^ cells in 0.1 ml of sterile 0.9% NaCl and 0.1 ml of Matrigel) were injected into the right and left mammary fat pads of six-week-old female nonobese diabetic/severe combined immunodeficient (NOD/SCID) mice (Jackson Laboratories, Bar Harbor, ME, USA). All procedures involving the mice were in accordance with the Guide for the Care and Use of Laboratory Animals and on a protocol approved by the Institutional Animal Care and Use Committee of the University of Pittsburgh.

**Figure 4:**
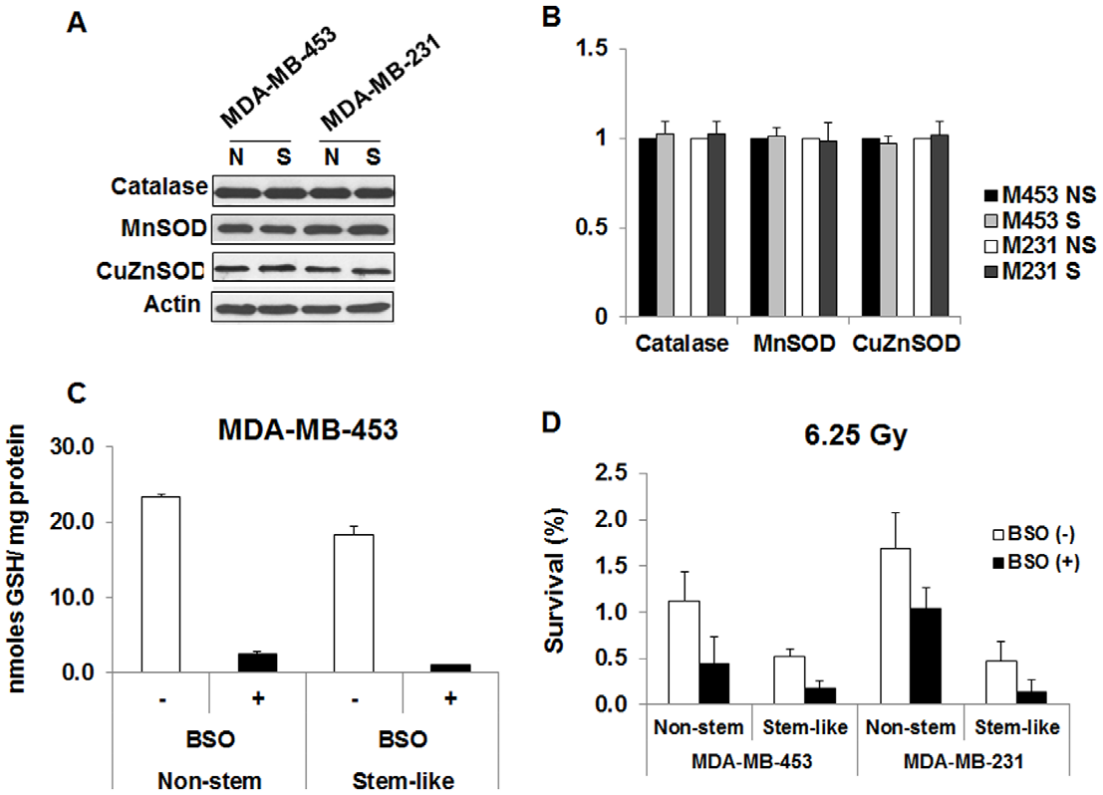
Role of anti-oxidant agents in radiosensitivity of non-stem cells and CSC-like cells. (**A**) Non-stem (N) and stem-like (S) MDA-MB-453 and MDA-MB-231 cells were harvested. Lysates containing equal amounts of protein (20 µg/ml) were separated by SDS-PAGE, and immunoblotted with anti-MnSOD, anti-CuZnSOD, or anti-catalase antibody. Actin was shown as an internal standard. (**B**) Densitometry analysis of each band was performed. The area integration of optical density of each band in stem-like cells (S) was compared with that in non-stem cells (NS). Error bars represent standard error from the mean for three separate experiments. (**C**) MDA-MB-453 non-stem cells and stem-like cells were treated with 200 µM L-buthionine-sulfoximine (BSO) for 24 hr and GSH content was determined. Error bars represent standard error from the mean for three separate experiments. (**D**) BSO-treated/untreated non-stem and stem-like MDA-MB-453 and MDA-MB-231 cells were irradiated at 6.25 Gy and survival was determined. Error bars represent standard error from the mean for three separate experiments.

### Reagents and Antibodies

L-Buthionine-sulfoximine (BSO), cycloheximide and aphidicolin were obtained from Sigma Chemical Co. (St. Louis, MO, USA). CP466722 was purchased from Selleck Chemicals (Houston, TX, USA). Anti-ATM, anti-phosphorylated ATM and anti-phosphorylated H2AX antibody were from Cell Signaling (Beverly, MA, USA). Anti-manganese-containing superoxide dismutase (MnSOD) was purchased from Millipore (Billerica, MA, USA). Anti-copper-zinc-containing superoxide dismutase (CuZnSOD) was from Stressgen (Farmingdale, NY, USA). Anti-catalase was from Epitomics (Burlingame, CA, USA).

### Fluorescence Microscopy

The morphological features and fluorescence signals for CSC-like and non-CSC cells were confirmed with phase contrast and fluorescence microscopy (Axiovert 40 CFL, Carl Zeiss Microimaging, NY, USA). The data were analyzed by microscope imaging processing software AxioVision from Zeiss.

**Figure 5:**
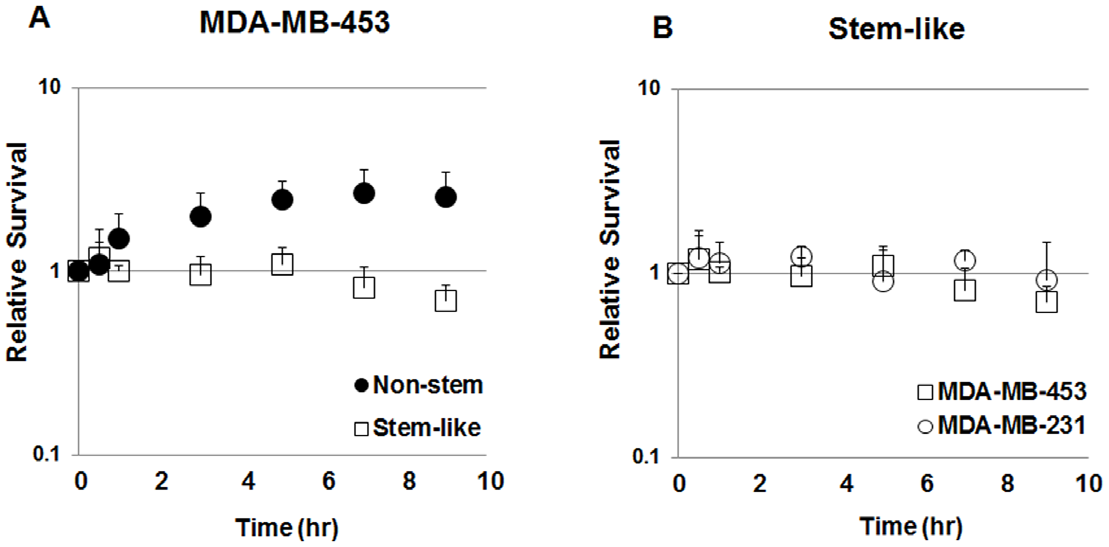
Analysis of sublethal damage repair. (**A**) Non-stem and stem-like MDA-MB-453 cells were exposed to two fractions of γ-radiation (5.0+2.5 Gy for non-stem and 3.75+2.5 Gy for stem-like) and incubated at 24°C for various time intervals between two exposures. Survival was compared to control group (irradiated without post-incubation) and plotted. Error bars represent standard error from the mean for three separate experiments. (**B**) Stem-like MDA-MB-453 and MDA-MB-231 were exposed to two fractions of γ-radiation (3.75+2.5 Gy) and incubated at 24°C for various time intervals between two exposures. Survival was compared to control group (irradiated without post-incubation) and plotted. Error bars represent standard error from the mean for three separate experiments.

**Figure 6:**
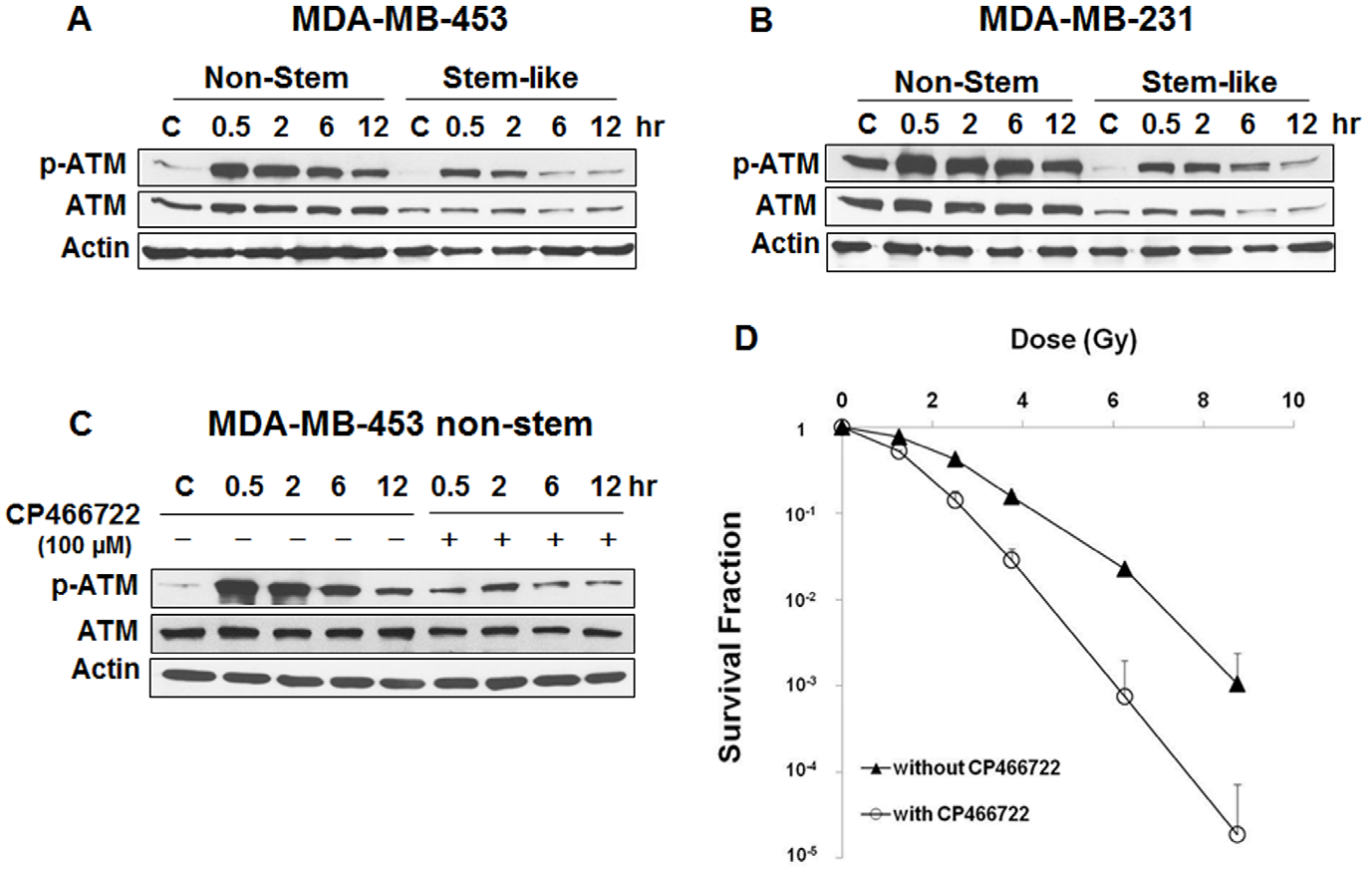
Ionizing radiation-induced phosphorylation of ATM and effect of ATM inhibitor CP466722 on radiosensitivity. (**A, B**) Non-stem and CSC-like MDA-MB-435 and MDA-MB-231 cells were irradiated at 8.75 Gy and phosphorylation (activation) of ATM was determined various times (0.5–12 hr) after irradiation. Lysates containing equal amounts of protein (20 µg) were separated by SDS-PAGE and immunoblotted with anti-ATM or anti-phospho-ATM antibody. Actin was shown as an internal standard. (**C**) MDA-MB-453 non-stem cells were pretreated with/without 100 µM CP466722 for 30 min, irradiated at 6.25 Gy and incubated various times before immunoblot analysis as described above. (**D**) MDA-MB-453 non-stem cells were pretreated with/without 100 µM CP466722 for 30 min, irradiated at various doses (1.25 Gy–8.75 Gy) and incubated for 6 hr before colony formation analysis. Error bars represent standard error from the mean for three separate experiments.

**Figure 7:**
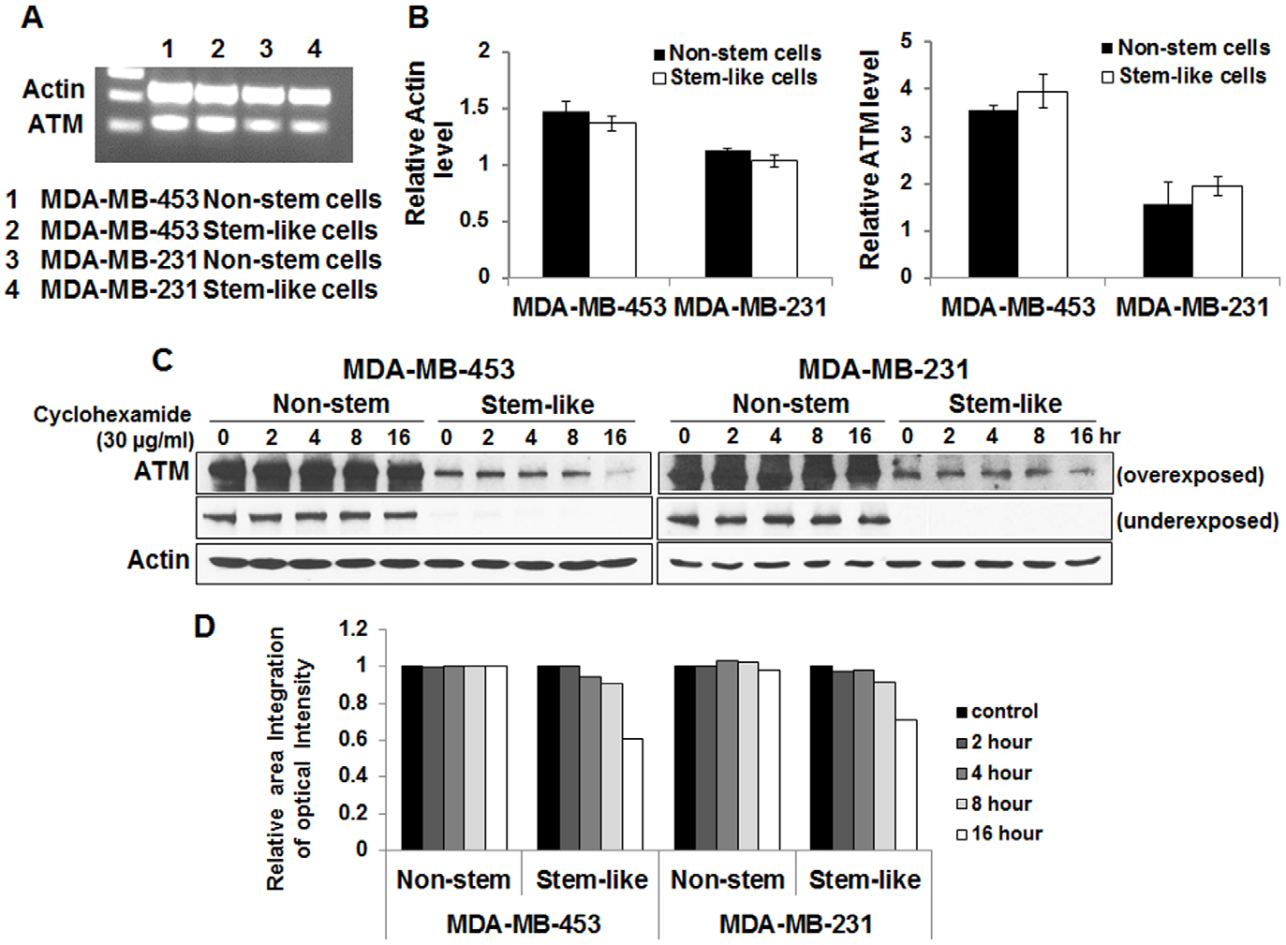
Determination of *ATM* gene expression and ATM protein stability in non-stem and CSC-like MDA-MB-435 and MDA-MB-231 cells. (**A, B**) Total RNA was extracted and reverse transcribed into cDNA. Human ATM mRNA was amplified and analyzed electrophoretically, and then quantified by Un-Scan-It gel software. (**C, D**) Cells were treated with 30 µg cycloheximide (CHM: >95% protein synthesis inhibition) for various times (2–16 hr) and harvested. Lysates containing equal amounts of protein (20 µg/ml) were separated by SDS-PAGE, and immunoblotted with anti-ATM or anti-actin antibody. Actin was shown as an internal standard. Densitometry analysis of each band was performed as described in Figure 4.

**Figure 8:**
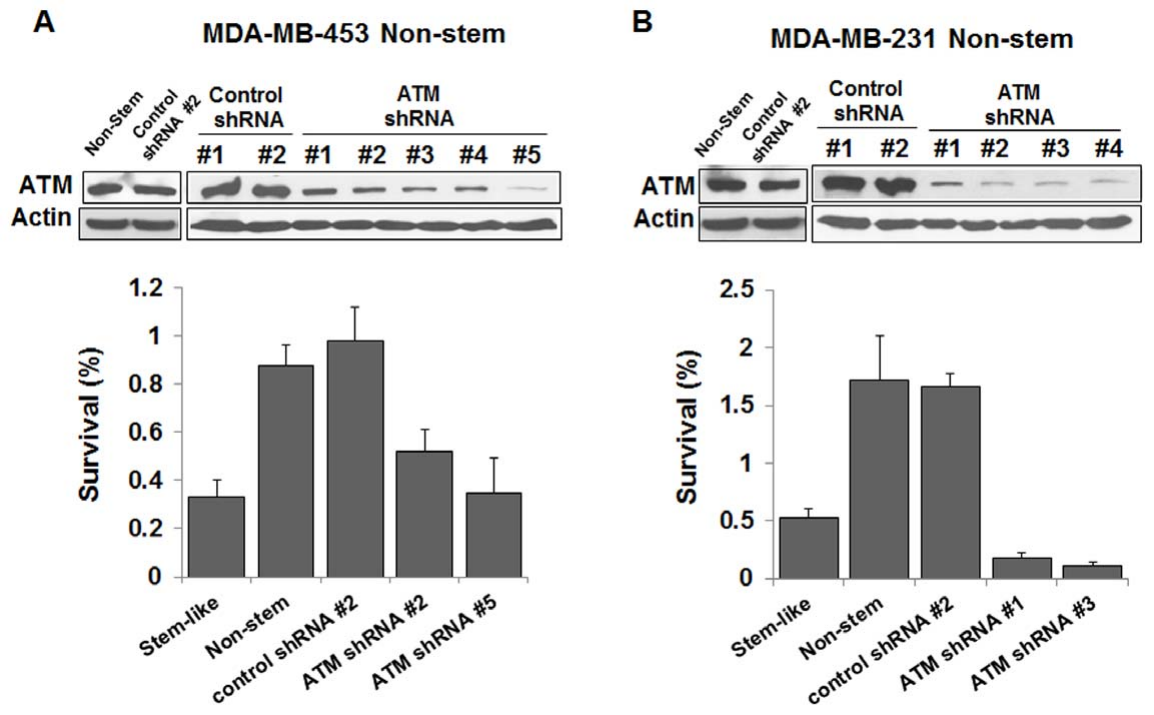
Role of ATM in radiosensitivity in non-stem MDA-MB-453 (A) and MDA-MB-231 (B) cells. Non-stem cells were infected with control shRNA or ATM shRNA lentiviral particle (2.5×10^4^–10^5^ IFU) and stable clones were selected by treatment with 10–100 µg/ml puromycin. ATM knockdown level was assessed by immunoblot assay as described in Fig. 6 (upper panels) and survival was determined after irradiation at 6.25 Gy (lower panels). Error bars represent standard error from the mean for three separate experiments.

### CD44 and CD24 Staining

Immunostaining of MDA-MB-453 cell lines was performed with an APC-labeled monoclonal antibody (mAb) against CD44 and a PE-labeled mAb against CD24 (BD Biosciences, Inc., Franklin Lakes, NJ, USA). Staining was performed with recommended protocols of the supplier. Analysis was performed using the FACScan flow cytometer, and results were analyzed with CellQuest software (both from Becton Dickinson Immunocytometry Systems, San Jose, CA, USA).

### Protein Extracts and PAGE

Cells were scraped with 1×Laemmli lysis buffer (including 2.4 M glycerol, 0.14 M Tris (pH 6.8), 0.21 M SDS, and 0.3 mM bromophenol blue and boiled for 5 min. Protein concentrations were measured with BCA protein assay reagent (Pierce, Rockford, IL, USA). The samples were diluted with 1×lysis buffer containing 1.28 M β-mercaptoethanol, and an equal amount of protein was loaded on 8–12% SDS-polyacrylamide gels. SDS-PAGE analysis was performed according to Laemmli [14] using a Hoefer gel apparatus.

### Immunoblot Analysis

Proteins were separated by SDS-PAGE, electrophoretically transferred to nitrocellulose membranes and blocked with 5% skim milk in TBS-Tween 20 (0.05%, v/v) for 30 minutes. The membrane was then incubated with antibodies against Oct 4, ATM, p-ATM (Cell Signaling, Danvers, MA, USA), or β-actin (Santa Cruz Biotechnology, Santa Cruz, CA, USA) for 1.5 hr. Horseradish peroxidase-conjugated anti-rabbit or anti-mouse IgG was used as the secondary antibody. Immunoreactive protein was visualized by the enhanced chemiluminescence protocol (ECL).

**Table 1:** Percentage of cells stained γ-H2AX positive after irradiation at 2.5 Gy.

MDA-MB-453	Non-stem	Stem-like
0 hr	10.0±4.7	12.3±0.6
0.5 hr	57.0±4.5	85.9±6.8
2 hr	30.0±4.1	70.7±9.4
6 hr	21.8±8.3	41.3±9.2
12 hr	10.4±4.8	13.3±9.1
**MDA-MB-231**	**Non-stem**	**Stem-like**
0 hr	12.7±3.3	11.1±4.0
0.5 hr	93.4±2.0	94.2±5.7
2 hr	57.9±9.4	85.2±5.9
6 hr	44.2±8.0	67.8±2.7
12 hr	26.0±9.1	34.8±6.9

Kinetics of γ-H2AX foci removal after irradiation. Non-stem and CSC-like MDA-MB-435 and MDA-MB-231 cells were irradiated at 2.5 Gy. Various times (0.5–12 hr) after irradiation, cells were fixed and immunostained with anti-phospho-H2AX antibody. Nuclei containing at least six fluorescent foci were considered positive and percentage of cells stained γ-H2AX positive was determined. Error bars represent standard error from the mean for three separate experiments.

**Table 2:** Percentage of cells stained γ-H2AX positive after irradiation at 8.75 Gy.

MDA-MB-453	Non-stem	Stem-like
0 hr	10.7±0.5	11.6±1.0
0.5 hr	92.5±7.6	96.6±2.5
2 hr	96±0.9	98±1.3
6 hr	75±3.4	88.4±2.4
12 hr	54.8±4.0	75.9±5.0
**MDA-MB-231**	**Non-stem**	**Stem-like**
0 hr	10.9±4.4	11.8±4.8
0.5 hr	92.2±0.7	92.9±0.3
2 hr	96.2±2.2	97.0±1.3
6 hr	89.3±4.2	92.5±0.5
12 hr	76.6±0.4	85.8±1.3

Kinetics of γ-H2AX foci removal after irradiation. Non-stem and CSC-like MDA-MB-435 and MDA-MB-231 cells were irradiated at 8.75 Gy. Various times (0.5–12 hr) after irradiation, cells were fixed and immunostained with anti-phospho-H2AX antibody. Nuclei containing at least six fluorescent foci were considered positive and percentage of cells stained γ-H2AX positive was determined. Error bars represent standard error from the mean for three separate experiments.

**Figure 9:**
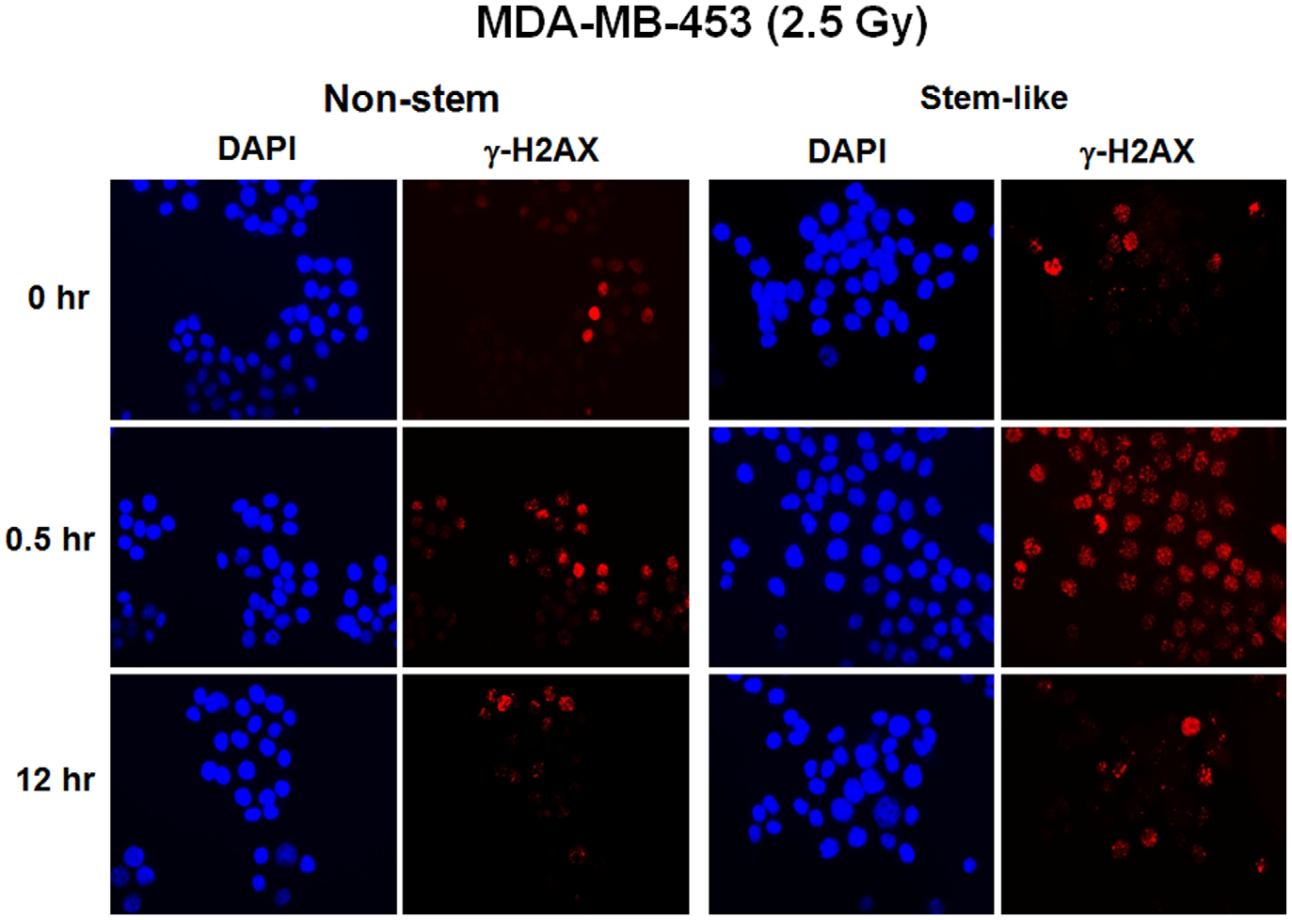
Ionizing radiation-induced γ-H2AX foci formation. Non-stem and CSC-like MDA-MB-453 cells were irradiated at 2.5 Gy. After 0.5 hr or 12 hr incubation, phosphorylated H2AX was detected by immunofluorescent staining with anti-phospho-H2AX antibody. Nuclei were stained with DAPI.

**Figure 10:**
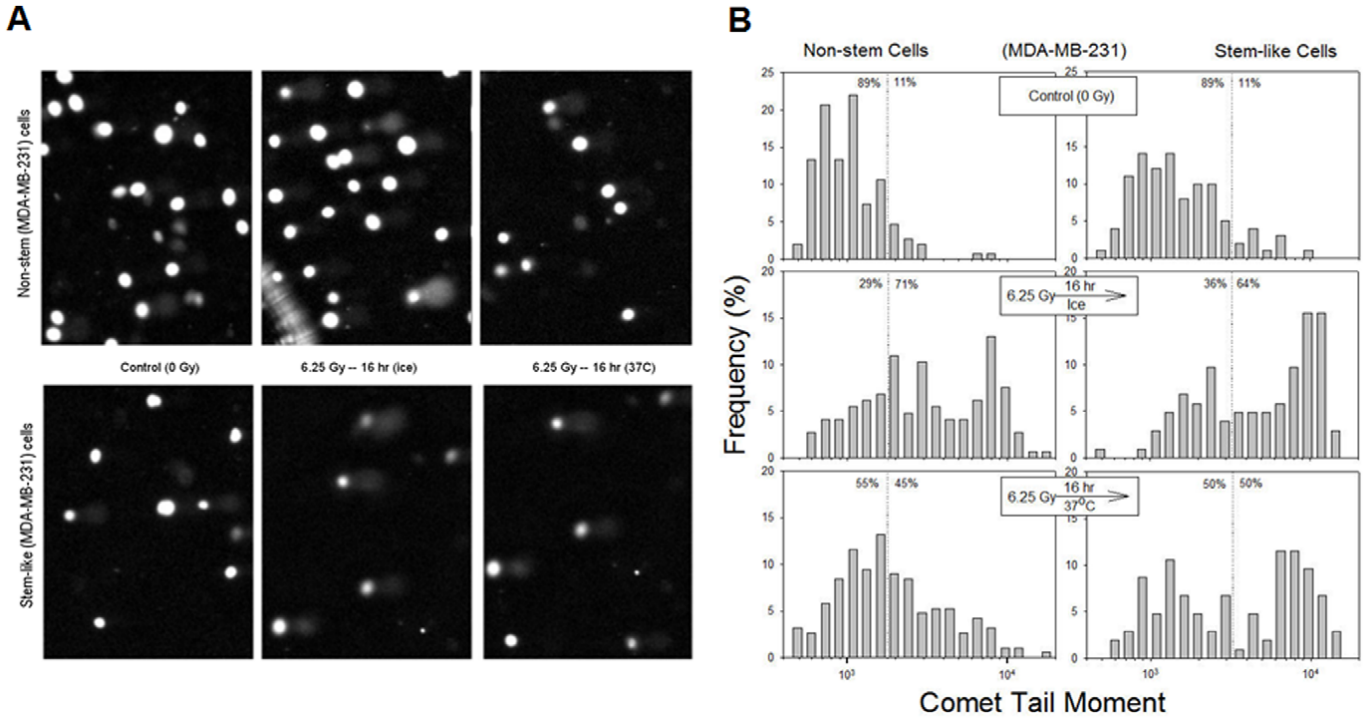
Alkaline comet images and their quantitative analysis for non-stem and stem-like MDA-MB-231 cells after irradiation. (**A**) Control (0 Gy) or irradiated (6.25 Gy) cells for both non-stem cells (upper row) and stem-like cells (bottom row) were subjected to alkaline comet assay (refer to the Methods) and the DNA was visualized by using a propidium iodide. (**B**) Distribution of comet tail moments for different treatments was plotted. Tail moments are the products of the distance of DNA migration (microns) and the amount of separated DNA (%).

### Densitometry Analysis

The Personal Densitometer SI from Molecular Dynamics was used to analyze the bands from immunoblotting assay. The ImageQuaNT program was used for the analysis.

### Colony Formation Assay

For colony formation assay, CSC-like and non-CSC cells were exposed to ionizing radiation, trypsinized, counted, and plated at appropriate dilutions (200–1×10^6^ cells/dish). The dishes were incubated at 37°C for 7–21 days to allow colony formation. Colonies were fixed, stained and counted manually. For every surviving fraction, the plating efficiency (PE) value was normalized.

### Cell Cycle Phase Distribution Analysis

We performed the cell cycle analysis according to company recommendations. Briefly, cells were trypsinized and centrifuged at 1500 rpm for 5 min, washed twice with PBS and then fixed with 70% cold ethanol. Fixed cells were stained using PI/RNase Staining Buffer (BD Bioscience) and incubated for 15 min at room temperature before analysis. Analysis was performed using the FACScan flow cytometer, and results were analyzed with CellQuest software (both from Becton Dickinson Immunocytometry Systems, San Jose, CA, USA).

### Measurement of Glutathione

Cells were washed twice with ice cold PBS, scraped into cold PBS, and centrifuged at 4°C for 5 min at 400×*g* to obtain cell pellets, which were frozen at −80°C. Pellets were then thawed and homogenized in 50 mM potassium phosphate buffer, pH 7.8 containing 1.34 mM diethylenetriaminepentaacetic acid. Total glutathione content was determined by the method of Anderson [15]. Reduced and oxidized glutathione were distinguished by addition of 2 µl of a 1∶1 mixture of 2-vinylpyridine and ethanol per 30 µl of sample followed by incubation for 1.5 hr and assay as previously described by Griffith [16]. All biochemical determinations were normalized to protein content using the method of Lowry *et al.* [17].

### Irradiation

Cells were grown in 60-mm Petri dishes, which were placed on a turntable located 4.8 cm from a ^137^Cs source in a vertical cylinder; dose rates during the period of these experiments were about 12 Gy/min.

### Knock Down of ATM in Nonstem Cells with shRNA Lentiviral Infection

Three different ATM shRNA lentivirus vectors were obtained from Santa Cruz Biotechnology (cat. # sc-29761-V, Santa Cruz, CA, USA) along with the appropriate control vector (cat. # sc-108080). The infection procedure was performed according to the instructions provided by the company. After infection, stable clones were selected by treatment with puromycin. ATM knockdown level was assessed by western blot assay.

### Immunostaining

After radiation, medium was discarded and cells were washed with 1×PBS buffer. Cells were fixed with 2% formaldehyde in PBS for 30 min at room temperature. Cells were washed again and added to 70% ethanol in PBS and then kept at −20°C in a freezer overnight. The next day, cells were blocked by using by 1% dry milk in PBS buffer containing Tween 20 and incubated for 30 min at room temperature. Cells were incubated with rabbit anti-γ-H2AX (Cell Signaling) primary antibody for 1 hr at room temperature. Following a wash in washing buffer (0.1% Tween 20 in PBS), the cells were incubated for 1 hr at room temperature in secondary antibody Alexa 555-conjugated goat anti-rabbit (Invitrogen, NY, USA) diluted in a 1∶500 ratio. Cells were washed with washing buffer, stained with 0.5 µg/ml DAPI for 1 min for counter staining, and then mounted with cover glass. Immunofluorescent staining was observed and photographed using a FLUOVIEW FV1000 CONFOCAL MICROSCOPE (Filters- ALEXA 555 and DAPI) and software FV10-ASW version 02.01.01.04 interfaced to an Olympus (Olympus, Center Valley, PA, USA).

### DNA Damage Assay

Ionizing radiation-induced DNA damage was assessed by the alkaline single-cell gel electrophoresis (“comet” assay) method. Cells were irradiated, trypsinized and embedded into 0.5% low-melting agarose on glass microscope slides. After treatment with alkaline lysis buffer, slides were subjected to electrophoresis, stained with propidium iodide (PI), and analyzed by epifluorescence microscopy. For quantification of DNA damage, fluorescence intensities (from PI staining) of the head and tail portions were obtained from each comet image, and the percentage intensity of the tail portion was multiplied by the length of the tail (in µm) (DNA migration) to yield a tail moment.

### Semi-quantitative Reverse Transcription-polymerase Chain Reaction Analysis

Total RNA was extracted and purified from cultured cells using the RNeasy Mini kit (Qiagen, Valencia, CA), according to the manufacturer’s instructions. The RNA was quantified by determining absorbance at 260 nm. Two µg of total RNA from each sample was reverse transcribed into cDNA using the High-Capacity cDNA Reverse Transcription kit (Life Technologies, Inc.) in a volume of 20 µl. Human ATM mRNA was amplified using the sense primer 5′-CGT GCC AGA ATG TGA ACA CC-3′ and the antisense primer 5′-ACA GTA GCA GCC AAG GAC AC-3′. Human actin mRNA was amplified using the sense primer 5′-CTG GGA CGA CAT GGA GAA AA-3′ and the antisense primer 5′-AAG GAA GGC TGG AAG AGT GC-3′. The polymerase chain reaction (PCR) was carried out as follows: 30 cycles of 94°C for 30 s, 55°C for 30 s, and 72°C for 60 s, followed by a 5-min extension stage at 72°C. Amplification products were analyzed electrophoretically on 1.0% agarose gel containing 0.1 µg/ml ethidium bromide and then quantified by Un-Scan-It gel software (Silk Scientific Inc.).

## Results

### Characterization of CSC-like Cells and Non-stem Cells

Blocked CSC-like cells can proliferate without differentiating and have characteristics of tumor-initiating cells [1]. This property arises as the result of stable transfection of the cells with a human Oct3/4 promoter driving the expression of GFP, although the mechanism of the block remains to be determined. In order to control for GFP expression, the corresponding non-CSC population was stably transfected with a plasmid expressing GFP under the control of a CMV immediate-early promoter [1]. Both CSC-like and non-CSC populations could be readily shown to express high levels of GFP whereas the untransfected population did not (Fig. 1A). Figure 1B shows that CSC-like cells were also highly enriched with CD44^+^ and CD24^−^ as previously described [1]. In addition, as shown in Figure 1C, both sets of CSC-like cells also selectively expressed octamer binding transcription factor 3/4 (Oct-4), which is known to maintain CSC-like properties [18]. Dontu et al. [19] reported that nonadherent mammospheres are enriched in cells having functional characteristics of the self-renewal potential of stem cells. As shown in Figure 1D, CSC-like cells, but not non-stem cells, formed mammospheres very well. Mammospheres of CSC-like cells were grown faster than those of non-stem cells and the difference of mammosphere size between CSC-like cells and non-stem cells was about 45 times on day 9. Similar results were also observed during xenograft tumor formation and tumor growth. As shown in Figure 1E, in comparison with non-stem cells, CSC-like cells formed tumors earlier and xenograft tumors grew faster. The average tumor size from CSC-like cells was 4.6-fold larger than that from non-stem cells 30 days after transplantation into 10 NOD/SCID mice.

### Comparison of Radiosensitivity of CSC-like Cells and Non-stem Cells

To determine the radiosensitivity of MDA-MB-453 and MDA-MB-231 CSC-like and non-CSC cells, we used colony formation assay following exposure to γ-rays (Fig. 2A) and survival curves were plotted (Figs. 2B and C). A final slope, D_0_ (the dose required to reduce the number of clonogenic cells to 37% of their former value), of the survival curve for each cell line was determined to measure radiosensitivity. D_0_ of CSC-like MDA-MB-453 cells and that of non-CSC MDA-MB-453 cells were 1.16 Gy and 1.55 Gy, respectively (Fig. 2B). As shown in Figure 2C, similar results were observed in MDA-MB-231 cells (0.94 Gy vs. 1.56 Gy). Our data clearly reveal that CSC-like cells are more sensitive to ionizing radiation than non-stem cells.

### Role of Cell Cycle Distribution in Differential Radiosensitivity of CSC-like Cells and Non-stem Cells

It has long been recognized that the degree of radiosensitivity is related to extrinsic factors (e.g., hypoxia) and intrinsic factors (e.g., cell cycle distribution, antioxidant levels, DNA repair capacity). We examined the role of these intrinsic factors in the differential radiosensitivity of CSC-like cells and non-stem cells. Early studies in radiobiology had revealed that cells are most radiosensitive during M and G_2_ phases and most resistant in late S phase [20]. We investigated whether cell cycle distribution plays a role in radiosensitivity. Cell cycle distribution was measured by measuring DNA content after staining with propidium iodide (PI). Figure 3A shows that S population was 36.2% and 41.5% in non-stem and CSC-like cells, respectively, in MDA-MB-453 cells. Figure 3B shows that, in MDA-MB-231 cells, S population was 27.3% and 22.8% in non-stem and CSC-like cells, respectively. These results illustrate that even though S-phase may contribute somewhat in the determination of radiosensitivity, it may not be a major factor. We further examined whether cell cycle effects contribute to radiosensitivity with synchronized cells. Treatment with 5 µM aphidicolin for 16 hr, which didn’t induce any significant cytotoxicity (data not shown), led to cell cycle arrest at the G_1_ phase (Fig. 3C). Asynchronized and synchronized cells were irradiated at 6.25 Gy and survival was determined (Fig. 3D). Figure 3D shows that synchronized CSC-like cells were still more sensitive to radiation than synchronized non-stem cells.

### Role of Antioxidants in Differential Radiosensitivity of CSC-like Cells and Non-stem Cells

Reactive oxygen species (ROS) are known to mediate the effect of ionizing radiation [21]. ROS are normally controlled by the antioxidant defense system including the tripeptide glutathione and antioxidant enzymes such as catalase, MnSOD (manganese-containing superoxide dismutase) and CuZnSOD (copper-zinc-containing superoxide dismutase). We examined whether antioxidant status is related to differential radiosensitivity of CSC-like cells and non-stem cells. We observed that the levels of antioxidant enzymes in non-stem cells and CSC-like cells were equivalent (Figs. 4A and 4B). These results suggest that the levels of antioxidant enzymes are an unlikely determinant of differential radiosensitivity. Next, we investigated the role of glutathione content, in particular the reduced form (GSH). We observed that only approximately 1% of the total glutathione exists in oxidized form (GSSG) (data not shown). Figure 4C shows that unlike antioxidant enzymes, the intracellular level of GSH in non-stem cells was 1.29-fold higher than that in CSC-like cells. To examine whether GSH plays an important role in differential radiosensitivity of CSC-like cells and non-stem cells, both cells were treated with 200 µM L-buthionine-sulfoximine (BSO) for 24 hr and GSH content was determined. BSO, an inhibitor of GSH synthase, reduced the intracellular level of GSH by 89% and 94% in non-stem cells and CSC-like cells, respectively (Fig. 4C). The level of GSSG was almost undetectable in BSO-treated cells (data not shown). BSO-treated and untreated control cells were irradiated at 6.25 Gy and survival was determined as shown in Fig. 4D. BSO treatment sensitized cells to radiation in non-stem cells as well as CSC-like cells. However, although BSO reduced GSH content by 89% in non-stem cells, survival of BSO-treated non-stem cells was similar or higher than that of untreated CSC-like cells at 6.25 Gy irradiation. These results suggest that GSH content plays an important role in radiosensitivity. However, GSH content may not be a requisite factor in differential radiosensitivity of CSC-like cells and non-stem cells.

### Role of DNA Repair Capacity in Differential Radiosensitivity of CSC-like Cells and Non-stem Cells

Previous studies have shown a good correlation between DNA repair capacity and radiosensitivity [3], [22], [23]. We hypothesized that DNA repair capacity is a determining factor for differential radiosensitivity of CSC-like cells and non-stem cells. We investigated this possibility by examining sublethal damage repair. It is a well-documented observation that mammalian cells have the ability spontaneously to recover from sublethal low LET (linear energy transfer) ionizing radiation-induced damage [24]. A fractionation technique is usually used to test for sublethal damage repair [25], [26]. For this study, we chose single doses at 1% isosurvival: 6.25 Gy for CSC-like cells and 7.5 Gy for non-stem cells in MDA-MB-453 cells (Fig. 2B). To determine the capacity of DNA damage repair, the radiation dose was divided into two fractions (3.75 Gy +2.5 Gy for CSC-like cells and 5 Gy +2.5 Gy for non-stem cells) separated by various time intervals (0.5–9 hr) at 24°C. Survival was determined after split-dose irradiation as shown in Fig. 5A. Figure 5A demonstrates that sublethal damage repair occurred in non-stem cells, but not in CSC-like cells. These data suggest an intrinsic difference between CSC-like cells and non-stem cells in terms of DNA repair capacity. This observation was confirmed in MDA-MB-231 CSC-like cells in which sublethal damage repair was also not observed after split-dose irradiation (Fig. 5B).

Previous studies have shown that ATM is responsible for sublethal damage repair [27], [28]. To examine the involvement of ATM in differential radiosensitivity of CSC-like cells and non-stem cells, cells were irradiated at 8.75 Gy and phosphorylation (activation) of ATM was determined at various times (0.5–12 hr) thereafter. Data from immunoblot analysis shows that ATM was rapidly phosphorylated within 0.5 hr and then gradually dephosphorylated in CSC-like cells as well as non-stem cells (Figs. 6A and 6B). However, activating phosphorylation of ATM was significantly higher in non-stem cells than in CSC-like cells in both cell lines. Moreover, intracellular level of total ATM protein in non-stem cells was 5–6-fold higher than that in CSC-like cells, indicating that difference in intrinsic level of ATM might be responsible for differential radiosensitivity. This possibility was examined by treating cells with ATM inhibitor CP466722. MDA-MB-453 non-stem cells were pretreated with 100 µM CP466722 for 0.5 hr and then irradiated at 6.25 Gy. After irradiation, cells were incubated at 37°C for various times (0.5–12 hr) before western blot analysis (Fig. 6C). As shown in Figure 6C, ionizing radiation-induced phosphorylation of ATM was inhibited by 77% following treatment with CP466722. CP466722 treatment was not cytotoxic (data not shown), however, it reduced D_o_ from 1.5 Gy to 0.98 Gy (Fig. 6D). Similar results were observed in MDA-MB-231 non-stem cells (data not shown). We expanded our observations to determine if the differences in the intracellular level of ATM are due to decreased *ATM* gene expression or ATM protein stability. Data from semi-quantitative RT-PCR assay shows no significant differences in *ATM* gene expression (Figs. 7A and 7B). However, ATM protein stability was somewhat different. Figures 7C and 7D show that ATM protein in CSC-like cells degraded faster than that in non-stem cells. This is probably due to differences in ubiquitination activity. We further investigated the role of ATM in radiosensitivity by using the small hairpin RNA (shRNA) technique for ATM knockdown. MDA-MB-453 and MDA-MB-231 non-stem cells were infected with lentiviral vectors containing either control shRNA or ATM shRNAs. After puromycin-resistant cell clones were selected, ATM protein knockdown was verified by immunoblotting (upper panels of Fig. 8). Figure 8 shows that expression of ATM was not changed by control shRNA, but effectively reduced by ATM shRNA in both non-stem cells. We obtained several stable clones and chose control shRNA #2 and ATM shRNA #2 and #5 in MDA-MB-453 non-stem cells (Fig. 8A) and control shRNA #2 and ATM shRNA #1 and #4 in MDA-MB-231 non-stem cells (Fig. 8B). For radiosensitivity assay, cells were irradiated at 6.25 Gy and colony formation assay was performed. Figure 8 shows that there was no significant change in radiosensitivity in control shRNA clones compared with non-stem cells. In contrast, non-stem cells with ATM knockdown were significantly more sensitive to ionizing radiation than control non-stem cells. These data suggest that ATM plays an important role in the differential radiosensitivity of CSC-like cells and non-stem cells.

It is well known that γ-phosphorylation of histone H2AX (γ-H2AX) “focus” formation is a rapid and sensitive cellular response to the presence of DNA double-strand breaks (DSBs) [29], [30]. H2AX is one of the targets of ATM phosphorylation and γ-H2AX foci formation after ionizing radiation reflects DNA damage and repair [31]. Figure 9 shows that γ-H2AX foci formation occurred rapidly within 0.5 hr after irradiation at 2.5 Gy and gradually reduced within 12 hr in both CSC-like and non-stem MDA-MB-453 cells. Nuclei containing at least six fluorescent foci were considered positive and kinetics of γ-H2AX foci removal after irradiation at 2.5 Gy or 8.75 Gy were analyzed in CSC-like cells and non-stem cells (Tables 1 and 2). As shown in Tables 1 and 2, the percentage of cells stained γ-H2AX reduced slowly in CSC-like cells in both cell lines. These results suggest that CSC-like cells have low DNA repair capacity which is responsible for the high radiosensitivity of these CSC-like cells. Similar results were observed with alkaline comet assay which detects DNA single- and double-strand breaks (Fig. 10A). After irradiation, 100–190 images were analyzed and % frequencies (linear) were plotted as a function of tail moments (logarithmic) (Fig. 10B). Dotted lines serve only to clarify the distributions, which is not to distinguish damaged or undamaged DNAs. When compared to non-stem cells (left column), stem-like cells (right column) show similar level of DNA damage (middle row), and the repair of DNA damage was blocked by ice (middle row) but progressed at 37°C (bottom row) (Fig. 10B). However, the efficiency of DNA repair appears to be much reduced for stem-like cells when compared to the repair of non-stem cells.

## Discussion

Several conclusions can be drawn upon consideration of the data presented here. First, CSC-like cells were more sensitive to ionizing radiation compared to their alternate subset non-stem cells. Second, although several factors have been known to determine cancer cell response to ionizing radiation, we observed that the main intrinsic determinant of differential radiosensitivity was DNA repair capacity, in particular ATM level, rather than cell cycle status or antioxidant levels.

Colony formation (clonogenic) assay is an *in vitro* cell survival assay based on the ability of a single cell to grow into a colony. The colony is defined to consist of at least 50 cells. The assay essentially tests every cell in the population for its ability to undergo “unlimited” division. This assay is the method of choice to determine cell reproductive death after treatment with ionizing radiation. It is well known that colonies from irradiated cells vary in size (Fig. 2A). This is probably due to radiation-induced cell cycle arrest. Interestingly, post-irradiated colonies appear to stain less densely than pre-irradiated colonies in non-stem cells. It is possible that an increased ATM activity promotes changes associated with epithelial-mesenchymal transdifferentiation (EMT) and results in increased cell mobility [32].

Early studies in radiation biology employed both *in vitro* and *in vivo* models to reveal that various determinant factors contribute to differential radiosensitivity. These factors are both extrinsic and intrinsic and include the tumor microenvironment (e.g., hypoxia and interaction with stromal elements) and radiation response elements (e.g., cell cycle distribution, antioxidant content, and DNA repair capacity) [3], [7], [33]–[35].

Iida et al. [36] revealed that hypoxia-induced cancer stem cell marker CD133 gene expression is mediated through OCT- and SRY(sex-determining region Y)-binding sites on P1 promoter. Oct-4 and SRY-box containing gene 2 (SOX-2) directly binds to OCT- and SRY binding sites, respectively, on P1 promoter and up-regulates hypoxia-induced promoter activity of CD133 gene expression. Our data in Figure 1C shows Oct-4 gene expression in both CSC-like cell lines. It is possible that Oct-4 may regulate cancer stem cell maker CD44 gene expression. This possibility needs to be further investigated.

The position of tumor cells within the cell cycle confers radiosensitivity. For instance, the late G_2_ and M phases are generally thought to be the most radiosensitive and the late S phase the most radioresistant. Al-Assar et al. [35] reported that breast cancer stem-like cells have a larger S-G_2_ fraction. Although cell cycle distribution may contribute somewhat to differential radiosensitivity, our data with synchronized cells suggest this to be a minor factor (Fig. 3D).

Antioxidants are well known to have a protective effect against radiation damage. MnSOD is considered to be one of the most important intracellular antioxidant enzymes and is localized to mitochondria. CuZnSOD is an intracellular enzyme mainly localized to cytosol. Catalase can decompose H_2_O_2_ which is made by living organisms exposed to oxygen, to water and oxygen. Data from Figure 4A shows no significant differences in the intracellular levels of antioxidant enzymes between CSC-like cells and non-stem cells. GSH is known to be the major ROS-scavenging system in cells. Nguyen et al. [37] observed an increased expression of genes involved in GSH synthesis in CSC suggesting that the intracellular level of GSH is responsible for radioresistance. Indeed, the lowering of endogenous GSH content by BSO treatment enhanced radiosensitivity in both CSC and non-stem cells ([3]; Figs. 4C and 4D). Nonetheless, data from Figure 4D shows that BSO-treated non-stem cells were still more resistant to radiation than BSO-treated CSC-like cells.

Bao et al. [3] demonstrated that radioresistance of glioma stem cells is mediated through preferential activation of the DNA damage checkpoint response and an increase in DNA repair capacity. Also, Ropolo et al. [8] observed that glioma stem cells display an elongated cell cycle and enhanced basal activation of checkpoint proteins that might contribute to their radioresistance. These studies suggest that radiosensitivity features are probably dynamic in nature. In this study, we observed a differential level of ATM in CSC-like cells and non-stem cells in MDA-MB-453 and MDA-MB-231 cell lines. Our data suggest that the intracellular level of ATM and/or its phosphorylation-dependent (activation) act as determinants of radiosensitivity. It still remains unclear, however, why non-stem cells have higher levels of ATM compared with CSC-like cells. At the present time, we can only speculate on the differential ATM gene expression in CSC-like cells and non-stem cells. Previous studies demonstrated that CSC-associated expression of genes such as *NOTCH* and *WNT* is responsible for radioresistance [3], [37]–[41]. In these CSC-like cells, CSC-associated gene expression may down-regulate *ATM* gene expression.

The studies presented here further elucidate the *ATM* gene regulation mechanisms involved in radiosensitivity. The differential radiation response in CSC-like cells and non-stem cells provides a useful model system for further investigation of this issue.
